# Tracheal intubating conditions in elderly patients when train-of-four count is zero after rocuronium 0.6 or 0.9 mg/kg. A secondary analysis

**DOI:** 10.1007/s10877-023-01012-6

**Published:** 2023-04-29

**Authors:** Matias Vested, Mian Hartoft, Lars S Rasmussen

**Affiliations:** Department of Anesthesia, Centre of Head and Orthopedics, University of Copenhagen, Section 6011, Rigshospitalet, Denmark

**Keywords:** Elderly patients, Tracheal intubating conditions, Neuromuscular blocking agents, Onset time

## Abstract

Purpose: The neuromuscular blocking agent rocuronium can be administered to facilitate tracheal intubation. We hypothesized that rocuronium 0.9 mg/kg provided a larger proportion of patients with vocal cords in abducted position compared to rocuronium 0.6 mg/kg at train-of-four (TOF) 0. Methods: This secondary analysis was based on 52 elderly surgical patients of which 36 patients received rocuronium 0.6 mg/kg and 16 patients received rocuronium 0.9 mg/kg. Neuromuscular block was monitored with acceleromyography with TOF stimulation at the ulnar nerve. The primary outcome was the proportion of patients with vocal cords in abducted position at TOF 0. Secondary outcomes were intubating conditions evaluated by the Fuchs-Buder scale, the Intubating Difficulty Score (IDS), onset time, and duration of action of rocuronium. Results: At TOF 0, a significantly larger proportion of patients had vocal cords in abducted position in the rocuronium 0.9 mg/kg group (81%) compared with the rocuronium 0.6 mg/kg group (53%); difference (%) 28, 95% Cl 3–53, P = 0.05. Excellent intubating conditions (Fuchs-Buder) were more common in the rocuronium 0.9 mg/kg group (62.5%); difference (%) 32.5, 95% Cl 4–61), P = 0.03. No significant difference was found in IDS or onset time of rocuronium (difference 19 s, 95% Cl: -5–43). Duration of action was significantly longer (difference 29 min, 95% Cl: 10–47) in the 0.9 mg/kg group. Conclusion: The proportion of patients with vocal cords in abducted position was significantly larger after rocuronium 0.9 mg/kg compared to rocuronium 0.6 mg/kg at TOF 0 monitored at the ulnar nerve.

## Background

During general anaesthesia the neuromuscular blocking agent (NMBA) rocuronium can be administered to facilitate tracheal intubation [1, 2]. With train-of-four (TOF) nerve stimulation at the ulnar nerve it is possible to determine onset time and duration of action of NMBAs [3]. However, monitoring the TOF response from the adductor pollicis muscle by ulnar nerve stimulation does not necessarily reflect the effect of NMBAs at the laryngeal muscles as they have different sensitivity to NMBAs [4, 5]. Rocuronium has a faster onset and recovery at the laryngeal adductor muscles compared with the adductor pollicis muscle, but the blockade is less intense [6]. This may influence tracheal intubating conditions as abduction of the vocal cords is essential.

In elderly patients both onset time and duration of action of rocuronium is prolonged compared with younger adults [7]. Increasing the dose of rocuronium in elderly patients reduces onset time, but at the expense of a longer duration of action [8]. Little is known about the effect of different doses of rocuronium on intubating conditions in elderly when TOF count is zero monitored at the ulnar nerve.

We have previously assessed tracheal intubating conditions, including position of the vocal cords, in patients above 80 years of age [7–9]. Patients received either rocuronium 0.6 mg/kg or rocuronium 0.9 mg/kg. In this secondary analysis, we aimed to compare tracheal intubating conditions after two different doses of rocuronium in elderly patients when there was no response to TOF stimulation at the ulnar nerve. We hypothesized that rocuronium 0.9 mg/kg would be associated with a larger proportion of patients with vocal cords in abducted position compared with rocuronium 0.6 mg/kg when TOF count was 0.

## Methods

**Fig. 1:**
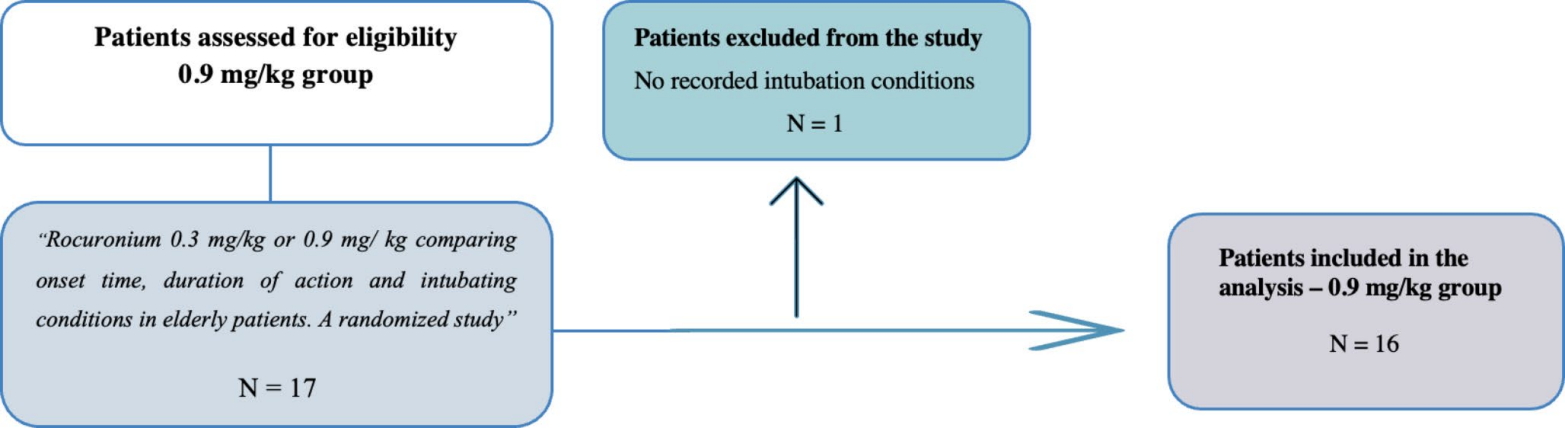
Flowchart of the included patients in the 0.9 mg/kg rocuronium group [8]

**Fig. 2:**
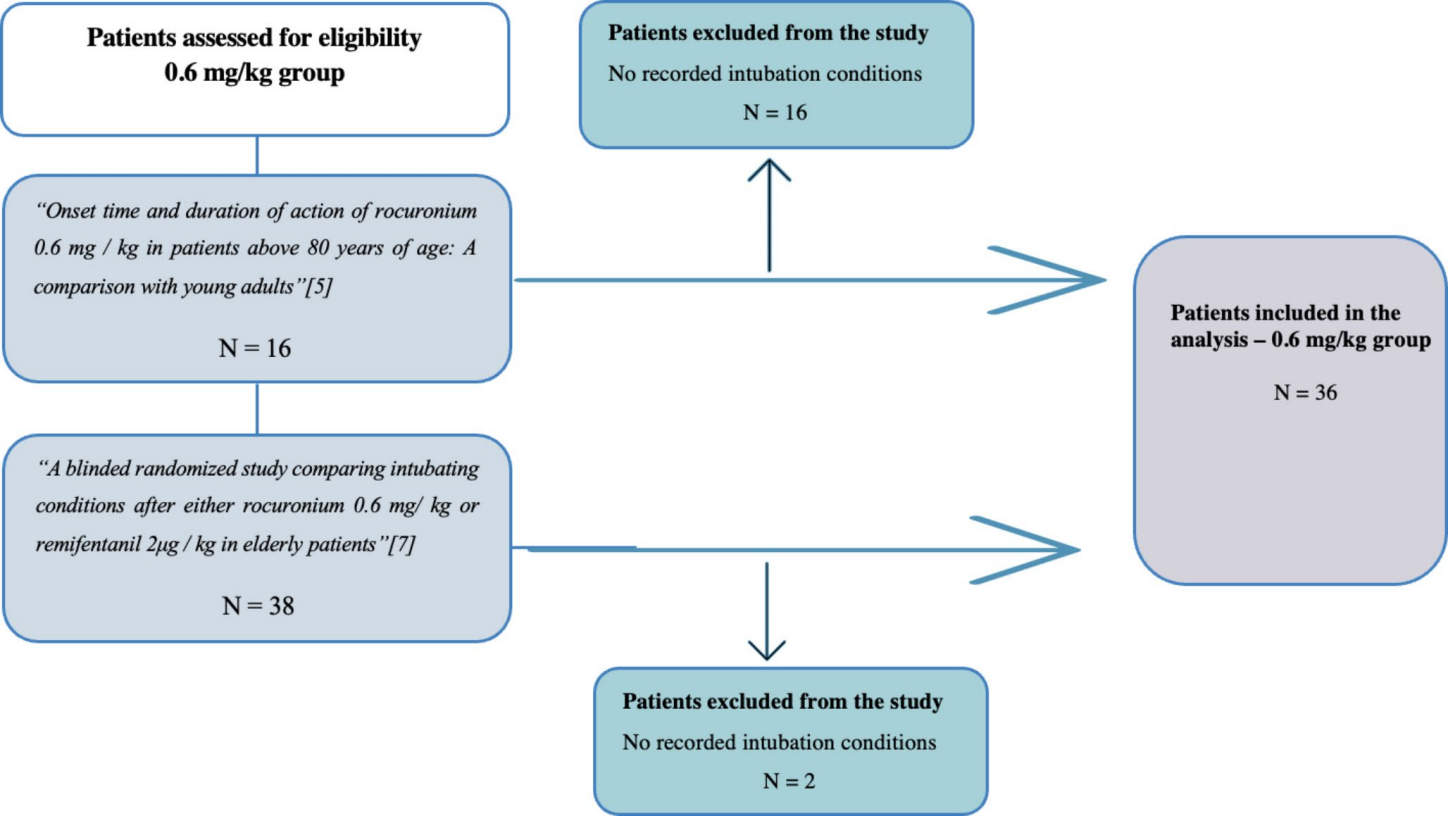
**-** Flowchart of the included patients in the 0.6 mg/kg rocuronium group

This study was a secondary analysis of data from three studies on patients receiving rocuronium 0.9 mg/kg or rocuronium 0.6 mg/ kg [7–9] (Figs. 1 and 2). The randomized studies were approved by the Danish Medicines Agency and the Scientific Ethics Committee. The protocol for the observational study [7] was submitted to the Danish Medicines Agency and the Ethics Committee, who decided that no formal approval was required. All three studies were approved by the Danish Data Protection Agency (Videnscenter for Dataanmeldelser) and conducted in accordance with the Declaration of Helsinki. Written informed consent was obtained from all patients. All three studies were registered at clinicaltrials.gov before enrollment of the first patient (NCT04287426, NCT04512313, NCT03857750). We included patients above 80 years with an American Society of Anaesthesiologists (ASA) physical health class I to III, scheduled for elective surgery with general anaesthesia and tracheal intubation using either rocuronium 0.9 mg/kg or rocuronium 0.6 mg/kg. Exclusion criteria were known allergy to rocuronium, neuromuscular disease interfering with neuromuscular monitoring or no recorded intubation conditions. Induction of anaesthesia comprised fentanyl 1.5-3 µg/kg and propofol 1–2 mg/kg and anaesthesia was maintained with an infusion of propofol of approximately 5 mg/kg/h and remifentanil 0.25–0.5 µg/kg/min.

Neuromuscular function was monitored by acceleromyograhpy with the TOF-Watch SX monitor connected to a computer [3]. The skin was cleaned before placing two ECG electrodes on the wrist over the ulnar nerve. The acceleration transducer was placed on the thumb and attached to a hand adaptor. The TOF Watch SX was started upon loss of eyelash reflex. Two TOF nerve stimulations were given followed by tetanic stimulation with 50 Hz for 5 s. Calibration was performed with the CAL button (CAL 2 modus) and neuromuscular function was monitored by TOF stimulation (2 Hz for 1.5 s) every 15 s.

Ideal body weight or actual body weight (the lowest) was used to guide the dosage of rocuronium. Ideal body weight was calculated in kg as height (cm) minus 105 for women and height (cm) minus 100 for men. Tracheal intubation was commenced as soon as the TOF count was 0. Tracheal intubating conditions including the position of the vocal cords were assessed by the Fuchs-Buder scale [3] and the Intubation Difficulty Score (IDS)[10] immediately after placement of the tracheal tube. Blinding in the two randomized studies was established by covering the TOF-Watch monitor, which ensured that the anaesthesiologist could not see the response at the nerve stimulator.

### Outcomes

The primary outcome was the proportion of patients with vocal cords in abducted position, according to the Fuchs-Buder scale [3]. Secondary outcomes were the proportion of patients with excellent intubation conditions evaluated by the Fuchs-Buder scale (defined as all qualities are excellent i.e. jaw relaxed, no resistance to laryngoscope, vocal cords with no movement and abducted, no movement of the patient and no coughing), intubating conditions evaluated by IDS, the proportion of patients with coughing during tracheal intubation, onset time of rocuronium defined as the time from the end of rocuronium injection to train-of-four (TOF) count of 0, and duration of action defined as time from end of rocuronium injection to reappearance of TOF ratio > 0.9.

### Statistical analysis

We used R studio software, Excel and Graphpad. Patient characteristics were reported as median with interquartile ranges (IQR) or count with frequencies. Categorical data were compared with Chi Square Test, while parametric data were compared with an unpaired t-test. Differences were calculated as the mean value with 95% confidence interval (CI). A P value < 0.05 was considered statistically significant. The sample size of 52 patients allowed us to detect a difference of 35% in the proportion with vocal cords in abducted position between the groups receiving rocuronium 0.9 mg/kg or rocuronium 0.6 mg/kg, respectively, with a power of 80%.

## Results

A total of 71 patients (54 patients in group rocuronium 0.6 mg/kg and 17 patients in group rocuronium 0.9 mg/kg) were eligible. Nineteen patients (18 in group rocuronium 0.6 mg/kg) had no tracheal intubating conditions recorded. Thus, a total of 52 patients were included for this study (Table 1).

**Table 1:** Characteristics of patients receiving either 0.6 mg/kg or 0.9 mg/kg rocuronium

	0.6 mg/kg rocuronium	0.9 mg/kg rocuronium
N Gender (female/male) Age (years) Height (cm) Weight (kg) BMI (kg/m^2^) ASA (I-II-III)	36 18/18 82.5 (81–84) 167 (163–178) 79 (61.8-85.75) 27.5 (22.9–29.3) 0/8/28	16 12/4 85 (84–86) 167 (160–174,2) 70 (63.5–75.5) 24.59 (22.3-27.05) 1/8/7

Median (IQR)

In group rocuronium 0.9 mg/kg a significantly larger proportion of patients had vocal cords in abducted position compared to group rocuronium 0.6 mg/kg with a difference of 28% (95% Cl, 3–53), P = 0.05 (Table 2). A significantly larger proportion of patients had excellent intubating conditions (Fuchs-Buder scale) after rocuronium 0.9 mg/kg with a difference of 32.5% (95% Cl, 4–61), P = 0.03 (Table 2).

**Table 2:** Tracheal intubation conditions (Fuchs-Buder) in patients receiving either 0.6 mg/kg or 0.9 mg/kg rocuronium

	0.6 mg/kg rocuronium	0.9 mg/kg rocuronium	Difference (%) with 95% CI	P-value
N	36	16		
Vocal cords position				
Abducted^a^ Intermediate Closed	19 (53%) 12 (33%) 5 (14%)	13 (81%) 3 (19%) 0	28 (3 to 53) 14 (-10 to 38)	0.11
Vocal cords movement				
None Moving Closing	30 (83%) 5 (14%) 1 (3%)	15 (94%) 1 (6%) 0	11 (-6 to 28) 8 (-8 to 24)	0.56
Reaction to intubation: movement				
None Slight Vigorous	34 (94%) 2 (6%) 0	16 (100%) 0 0	6 (-2–14)	0.86
Reaction to intubation: coughing				
None Slight Sustained	24 (66%) 11 (31%) 1 (3%)	13 (81%) 1 (6%) 2 (13%)	15 (-10 to 40) 25 (6 to 44) 10 (-7 to 27)	0.08
Laryngoscopy: jaw relaxation				
Relaxed Not fully Poor	31 (86%) 5 (14%) 0	15 (94%) 1 (6%) 0	8 (-8 to 24) 8 (-8 to 24)	0.75
Laryngoscopy: resistance to laryngoscope				
None Slight Reactive	31 (86%) 5 (14%) 0	15 (94%) 1 (14%) 0	8 (-8 to 24) 8 (-8 to 24)	0.75
Intubating conditions				
Excellent^b^ Good Poor	11 (30%) 19 (53%) 6 (17%)	10 (62.5%) 4 (25%) 2 (12.5%)	32.5 (4 to 61) 28 (1 to 55) 4.5 (-15 to 25)	0.08

Data presented as count and frequencies. P-values based on chi square test, a: P = 0.05, b: P = 0.03

No difference was found in onset time comparing group rocuronium 0.9 mg/kg with group rocuronium 0.6 mg/kg with mean difference of 19 s (95% Cl: -5–43). Duration of action was significantly longer after rocuronium 0.9 mg/kg with a mean difference of 29 min (95% Cl: 10–47), P = 0.03, (Table 3).

**Table 3:** Tracheal intubation conditions (Intubation Difficulty Scale), onset time and duration of action in patients receiving either 0.6 mg/kg or 0.9 mg/kg rocuronium

	0.6 mg/kg rocuronium	0.9 mg/kg rocuronium	Difference	P-value
N	36	16		
Onset time, s	127 (39)	108 (40)	19 (-5 to 43)	0.16
Duration of action, min	86 (24)	115 (42)	29 (10 to 47)	0.03
Intubation Difficulty Scale				0.24
- Easy (0) - Slight (0 < IDS ≤ 5) - Moderate/major (5 < IDS)	9 (25%) 22 (61%) 5 (14%)	6 (37.5%) 10 (62.5%) 0	12.5(-15 to 40) 1.5 (-27 to 30)	

Data presented as count/frequencies and mean (SD). P-values based on chi square and unpaired t-test

No difference in IDS score or proportion of patients with coughing during tracheal intubation was found (Table 3).

## Discussion

When TOF count was zero at the ulnar nerve a significantly larger proportion of elderly patients in group rocuronium 0.9 mg/kg had vocal cords in abducted position compared to group rocuronium 0.6 mg/kg. Excellent intubating conditions were also achieved in a significantly larger proportion of patients administered rocuronium 0.9 mg/kg.

The strengths of our study include the randomized blinded design in two of the studies with blinding of the anaesthesiologists performing the intubation. Neuromuscular monitoring was performed according to research guidelines, which minimized the risk of imprecise data [3]. The studies had the similar inclusion and exclusion criteria, which made it reasonable to combine the data. However, there are also some limitations to this study. We did not collect data regarding the Fuchs-Buder scale in the patients from the observational study [7] with administration of rocuronium 0.6 mg/kg. Hence, data from this study [7] could only be used in the analysis of some of the secondary outcomes. This may explain why we did not find a difference in all outcome variables on the Fuchs-Buder Scale (Table 2) as there could be a risk of a type two error. Also a type two error possibly can explain the lack of difference in onset time since our sample size based on proportion of patients with vocal cords in abducted position may have been too small to detect a significant difference in onset time. In this matter a study in young adults (mean age of approximately 30 years) with a larger sample report significant difference in onset time comparing 0.6 and 0.9 mg/ kg of rocuronium [11]. Finally, intubating conditions were assessed by different investigators, and this can increase variability and lead to an inability to detect a difference.

We did not include data on postoperative hoarseness and sore throat. Therefore, we cannot conclude whether there is association between the presence of abducted vocal cords and postoperative hoarseness and sore throat. Injuries may occur during insertion of the tracheal tube if the vocal cords are more or less closed [12]. However, our sample was relatively small and we may not have been able to detect such difference.

Vocal cords were in abducted position in 81% after administration of rocuronium 0.9 mg/kg and almost two thirds had excellent intubating conditions. After administration of rocuronium 0.45 mg/kg another study [2] found that 80% of the patients had excellent intubation conditions while we found that 30% had excellent intubating conditions after administration of rocuronium 0.6 mg/kg. Finally, a recent Japanese study on elderly patients found excellent intubation conditions in all patients administered rocuronium 1 mg/kg as opposed to our study with excellent intubation conditions in approximately two thirds of the patients receiving rocuronium 0.9 mg/kg. Also rocuronium 0.6 mg/kg resulted in poor intubating conditions in almost 50% of the cases [13] whereas we found poor intubating conditions in 17% of the cases. This illustrates the variation in the assessment of intubation condition even though the same rating scale is used and calls for careful training. A clinical consequence of our finding could be that time to placement of the tracheal tube could be prolonged if the attending anaesthesiologist decided to await full effect of rocuronium on the laryngeal muscles. Time to tracheal intubation (i.e. from taking the laryngoscope until tracheal intubation was verified by capnography) after administration of 0.6 mg/kg of rocuronium was 195 s [7] whereas time to tracheal intubation was approximately 70 s after administration of 0.9 mg/kg [8]. However, time to tracheal intubation was not a predefined outcome of this study.

Our study findings illustrate the difference in sensitivity towards rocuronium between striated muscles in the hand and in the larynx. The findings are relevant for clinicians because the difference in intubating conditions were detected despite that a TOF count of zero was achieved by both doses (i.e. 0.6 and 0.9 mg/kg). Therefore, a TOF count of zero at the ulnar nerve in this elderly patient population did not accurately predict the optimal timing for tracheal intubation. Nevertheless, a dose of 0.9 mg/kg of rocuronium provides better intubating conditions, but this is at the cost of a prolonged duration of action.

The muscles around the eye can be used as an alternative to neuromuscular monitoring at the ulnar nerve. The response of m. corrugator supercilii reflects blockade of laryngeal adductor muscles [13]. Monitoring neuromuscular blockade at the corrugator supercilii can therefore reflect rocuronium neuromuscular blockade at the laryngeal muscles and help the clinician in the timing of tracheal intubation in terms of abducted vocal cords [14]. However, clinicians should be familiar with this technique also to ensure correct detection of full recovery of the neuromuscular blockade before awakening the patient.

There is no standardized consensus on how to evaluate intubating conditions [1]. In this study both the Fuchs-Buder scale [3] and the IDS score [10] were used to evaluate the intubating conditions. The Fuchs-Buder scale [3] focuses on the effect of NMBAs on the laryngeal muscles, whereas the IDS score [10] also assesses number of intubation attempts and the technique employed. Our primary endpoint was the proportion of patients with vocal cords in abducted position and that was significantly different between groups along with the proportion of excellent intubating conditions. In Table 2 we report all three categories for position of the vocal cords along with all three qualities of the items at the Fuchs-Buder scale and the Chi Square tests are therefore based on each of these three categories.

In conclusion, at TOF 0 monitored at the ulnar nerve the proportion of patients with vocal cords in abducted position was significantly higher in patients who received rocuronium 0.9 mg/kg compared to the patients who received rocuronium 0.6 mg/kg.

## Declarations

### Trial registration number

A secondary analysis of three trials: NCT04287426, NCT04512313, NCT03857750.
